# A sewing needle in the liver in children: A case report and literature review

**DOI:** 10.1097/MD.0000000000029339

**Published:** 2022-07-08

**Authors:** Huiwu Xing, Bingqian Tan, Chenyu Yang, Mingman Zhang

**Affiliations:** Department of Hepatobiliary Surgery, Children’s Hospital of Chongqing Medical University, Chongqing Key Laboratory of Pediatrics, National Clinical Research Center for Child Health and Disorders, Ministry of Education Key Laboratory of Child Development and Disorders, Chongqing, China.

**Keywords:** case report, children, intrahepatic needles, literature review, management

## Abstract

**Rationale:**

In clinical practice, foreign bodies (FBs) in the digestive tract are more common in children, but intrahepatic FBs are rare, especially those that can cause infection, bleeding, bile leakage, and other complications. However, there is no consensus on its diagnosis and treatment due to the lack of large-scale cohort studies.

**Patient concerns:**

Case 1 is a 4-years 8-months-old girl, who at the age of 10 months, showed an X-ray finding of a striped FB in her liver, with no symptoms. However, the patient’s parents refused surgery. After nearly 4 years of active surveillance, the patient visited our hospital for surgery. Case 2, a 2-year-old male, reported a sewing needle that completely pierced into the right upper abdomen due to an accidental fall that took place half-a-day before admission. He only had right upper abdominal pain. CT showed a striped FB in the liver.

**Diagnosis:**

FB in the liver (sewing needle).

**Interventions:**

Both the patients were injected with human tetanus immunoglobulin and underwent surgical removal.

**Outcomes:**

Both patients recovered smoothly and had no complications during follow-up.

**Lessons:**

Active surveillance might be considered for cases with no symptoms or complications and no displacement of the FB, but surgery should be the first choice. If the patient’s condition is complicated, it is recommended to use ultrasound or X-ray to help decision-making during the operation. Additionally, tetanus, child safety, and family education are important supportive measures.

## 1. Introduction

Patients with foreign bodies (FBs) in the liver are very rare in clinical practice, and most of them are children, elderly individuals, psychiatric patients, and alcoholics.^[1,2]^ Neglected and radiolucent FBs can lead to long-term discomfort and serious complications.^[3]^ In addition, pediatric cases with a sewing needle in the liver are very rare and differ from adult cases, such as the route of the needle into the liver. Only 10 pediatric cases with needles in the liver have been reported in PubMed, and there is no consensus regarding diagnosis and treatment. Children are more vulnerable to school violence, domestic abuse, and neglect, and more attention should be paid to the prevention of these accidents. Here, we report our experience and review the literature to provide a reference for the diagnosis and treatment of pediatric patients with a sewing needle in the liver.

## 2. Consent

This study was reviewed and approved by the Institutional Review Board of the Children’s Hospital of Chongqing Medical University. Written informed consent was obtained from the legal guardians of the patients.

## 3. Case presentation

### 3.1. Case 1

**Figure 1.**
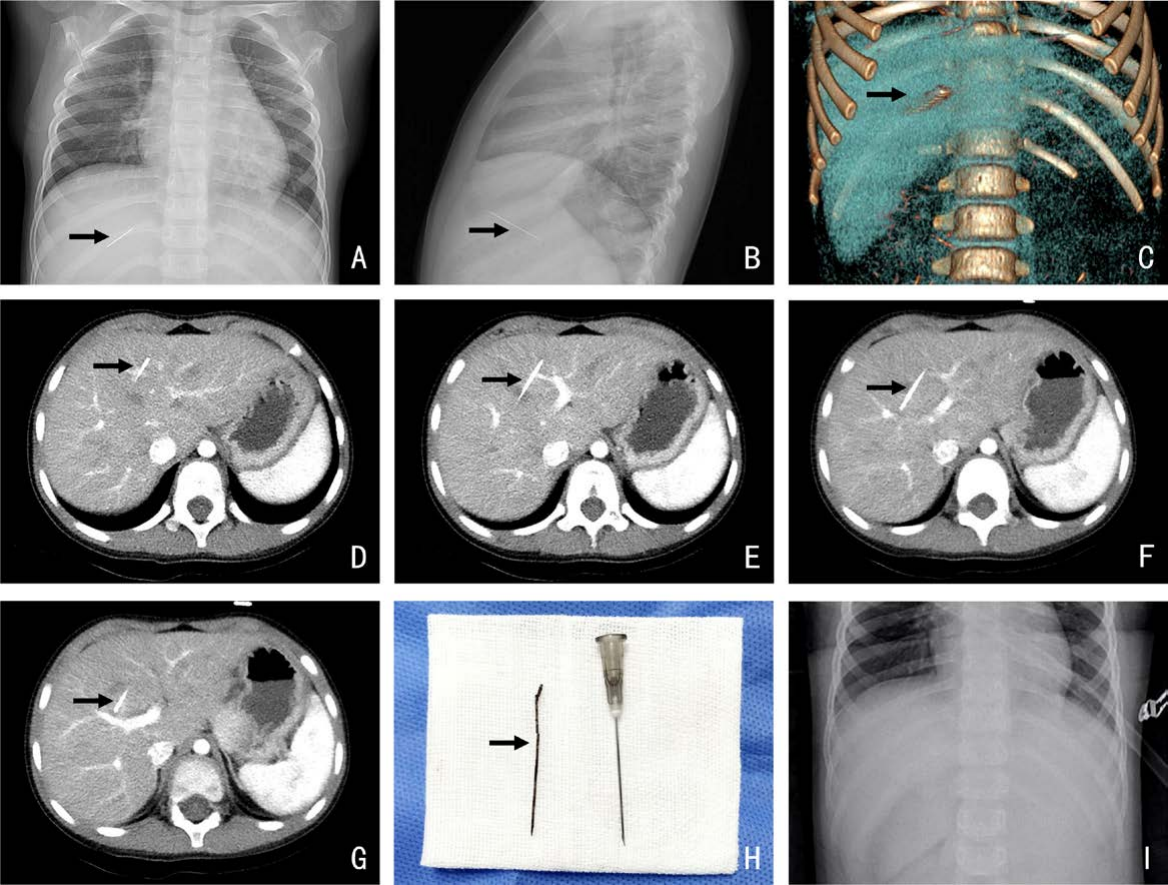
Preoperative and intraoperative images of case 1. Notes: The black arrow indicates FB. (A, B) Preoperative radiograph showing a high-density shadow in the hepatic region. (C) Preoperative CT reconstructed images showing a striated FB in the liver. (D, G) Contrast-enhanced CT showing the location of the striated FB in the liver before surgery. (H) The sewing needle is removed from the liver. (I) Intraoperative radiography showing that the FB had been removed.

A 4-year- 8-month-old girl-who at the age of 10 months, showed an X-ray finding of a striped high-density shadow in the hepatic region, but she had no symptoms. Interestingly, there was no FB on the radiograph 2 months prior, and her parents denied a history of sewing needle swallowing or trauma. The patient’s parents refused surgical removal because the child was too young. Therefore, medical treatment was limited to injection of human tetanus immunoglobulin. During the active surveillance period, the child regularly underwent radiography or CT, and no FB displacement or symptoms were found. When the patient visited our hospital at the age of 4 years and 2 months, her X-ray (Fig. 1A, B), and contrast-enhanced CT (Figure 1C–G) showed that a striped FB in the liver (approximately 3.8 cm long) was suspected to be a sewing needle; which pierced obliquely into the liver and pointed to the boundary between hepatic segments Ⅴ and Ⅶ; its tail was in hepatic segment ⅣA, near the sagittal part of the portal vein, which was approximately 3.2 cm from the body surface; and its tip was in hepatic segment Ⅷ, near the right anterior branch of the portal vein. Laboratory test results were normal. However, there were still risks of bleeding, infection, bile leakage. As the child grew up and the amount of exercise increased, so did the risk of developing complications. Therefore, at the age of 4 years and 8 months, the girl underwent laparotomy. During the operation, no scar on the liver surface, no adhesion to the peritoneum, and no trace of digestive tract perforation were found. With ultrasound guidance, the liver was cut longitudinally 1.5 cm to the right of the round ligament, and a rusty sewing needle approximately 4 cm long was found and completely removed (Fig. 1H). Intraoperative radiography revealed that the FB had been removed. No bile leakage was observed, and only a small amount of bleeding was observed during the operation. The child recovered satisfactorily and was discharged 4 days after the operation. No complications occurred during the follow-up of 21 months.

### 3.2. Case 2

A 2-year-old boy reported with a sewing needle that had completely pierced into the right upper abdomen because of an accidental fall that took place half-an-hour before the admission. A pinhole-like wound was observed on the skin surface in the right costal region, and he had pain in the right upper abdomen, but no other symptoms. Radiography showed a striped, high-density shadow in the right upper abdomen. CT showed a striped FB (approximately 3.5 cm) from the right abdominal wall to the right lobe of the liver. The laboratory test results were normal. The patient underwent emergency surgery. We found that the needle pierced the hepatic segment VI, and the tail was left in the abdominal wall. The needle (rust-free) was completely removed, and the liver wound was sutured. There was no bile leakage. He was given human tetanus immunoglobulin immediately after surgery. The patient recovered satisfactorily, and was discharged smoothly 2 days after the operation. No complications occurred during the 11 years of follow-up.

## 4. Discussion

Children are curious and have a poor ability to take care of themselves, especially infants, so there is a higher incidence of accidents such as swallowing FBs or accidental stab wounds. FBs in the liver are rare in clinical practice, and a literature review showed that FBs in the liver include fish bones (33%), toothpicks (27.3%), chicken bones (12.5%), and needles (9.1%).^[4]^ There are 3 main routes to the liver for FBs: (1) penetrating into the liver through the abdominal wall (skin), (2) migration from the gastrointestinal tract (GIT), and (3) via the bloodstream.^[5]^ However, to our knowledge, no case of a needle in the liver via the bloodstream has been reported. Studies have shown that the most common perforation sites in patients with FBs in the liver are the stomach, duodenum, and colon; however, gastrointestinal perforation caused by swallowing FBs is very rare, with an incidence of <1%.^[4,6]^ We conducted a literature search in PubMed about pediatric patients with the sewing needle in the liver, and only 12 cases were reported, including our cases (Table 1). Most patients were male (9/12, 75.0%) and under 3 years old (9/12, 75.0%), and the routes to the liver included the abdominal wall and GIT (50% vs 41.7%). Therefore, sewing needles in the liver rarely occur in children, most of whom are infant boys, and the main causes are stab wounds and swallowing.

**Table 1:** 12 pediatric cases with a needle in the liver.

Reference	Sex	Age	Cause	Symptom	Complication	Blood biochemistries	Route into liver	Location	Treatment	Outcome
Abel RM, et al 1971^[7]^	Male	11 months	Swallow	Cough, coryza and fever	None	Elevated WBC and blood lead level	Stomach	Left lobe	Laparotomy	Smooth recovery
Stone RK, et al 1976^[8]^	Male	6 months	Fall caused by abuse, doubtfully	A swelling in the right upper abdomen	None	Not described	Skin	Left lobe	Laparotomy	Smooth recovery and good prognosis
Crankson SJ. 1997^[9]^	Male	2 years	Unknown	None	None	Not described	Unknown	Right lobe	No intervention	Good prognosis
Nishimoto Y, et al 2003^[1]^	Male	1 year	Unknown	None	None	Normal	Skin, doubtfully	Left lobe	Laparotomy	Smooth recovery
Azili MN, et al 2007^[10]^	Female	14 years	Swallow	Abdominal pain and mild fever	None	Increased WBC and higher erythrocyte sedimentation rate	Stomach	Right lobe	Laparotomy	Smooth recovery and good prognosis
Avcu S, et al 2009^[11]^	Female	16 years	Swallow	Abdominal pain, nausea, and vomiting	Liver abscess	Elevated WBC, C-reactive protein, transaminases, and erythrocyte sedimentation rate	GIT	Right lobe	Laparotomy	Smooth recovery
Akçam M, et al 2009^[12]^	Male	5 years	Swallow	None	None	Normal	Duodenum	Right lobe	Endoscopic surgery	Smooth recovery
Saitua F, et al 2009^[13]^	Male	3 months	Accident	Cough and minor respiratory difficulty	None	Not described	Skin	Right lobe	Laparotomy	Smooth recovery and good prognosis
Dominguez S, et al 2009^[14]^	Male	3 years	Swallow, doubtfully	None	None	Normal	Duodenum	Hepatic segmentⅠ	Laparoscopic surgery	Smooth recovery and good prognosis
Xu BJ, et al 2013^[2]^	Male	5 months	Unknown	Mild respiratory symptoms	Infection	Increased WBC and platelet, and mild elevation of transaminase	Skin	Right lobe	Laparotomy	Smooth recovery and good prognosis
Our case 1	Female	4 years and 8 months	Accident, doubtfully	None	None	Normal	Skin	Hepatic segmentⅣandⅧ	Laparotomy	Smooth recovery and good prognosis
Our case 2	Male	2 years	Accident	Abdominal pain	None	Mild elevation of WBC and transaminase	Skin	Right lobe	Laparotomy	Smooth recovery and good prognosis

Children have a poor ability of expression, especially infants, so pediatric patients may express their physical discomfort only by crying. Moreover, needle wounds are often subtle, and most patients have only short-term nonspecific symptoms or even no symptoms (Table 1). Therefore, this method is difficult to detect. Caregivers often give comfort only and do not pay more attention to it. In addition, stab wounds or swallowing FBs may also occur in incidents, such as family abuse and school violence, and children may try to hide such conditions. Finally, these may lead to many complications, such as long-term retention of the FB in the liver or hepatic abscess, which influence the outcome and prognosis. We found that routine laboratory tests in most patients could not provide any specific diagnostic information (6/9, 66.7%; Table 1). The diagnosis of intrahepatic FBs mainly depends on imaging examinations, especially in patients who deny or forget swallowing FBs or trauma.^[10]^ Ultrasound is often the first choice for pediatric patients because of its low price, lack of radiation exposure, and ease of access in most hospitals, including primary health care institutions. However, CT is more useful for the diagnosis and decision of the treatment plan because of its higher resolution, better visibility, and understandability, which are helpful for localization.^[4]^ Furthermore, contrast-enhanced CT has important value for the determination of treatment plans because it can provide more information on the relationship of the FB with the surrounding organs and vessels.^[15]^

Similar to other FBs in the liver, the treatment decisions of patients with a needle in the liver consider the material, position, and displacement of the needle as well as the presence of symptoms and complications. We found that most patients underwent surgery (11/12, 91.7%), and all of them had good outcomes (12/12, 100%), which suggests that most patients had high surgical compliance and that the surgery was safe and effective (Table 1). However, because the needle was small and easily wrapped by tissues, most patients had no symptoms or complications (10/12, 83.3%; Table 1). *Senol A* et al reported that a patient had been asymptomatic for up to 6 years,^[16]^ our case had no symptoms for nearly 4 years, and *Crankson SJ* reported that 1 pediatric patient who received noninvasive treatment had no symptoms for 3 years^[9]^ (Table 1). Temporary active surveillance supported by first-visit or primary health care institutions rather than invasive treatment might be considered for cases with the absence of symptoms and complications and no displacement of the FB.^[9,11,13,14,16]^ However, with the growth of children, exercise, and the growth of the liver, the risk of complications also increases, and early surgical treatment may be the best choice for a better prognosis, which may reduce the difficulty of removing FBs.^[4]^ Laparotomy, laparoscopy, or endoscopic surgery can be performed according to the patient’s condition and medical technology level.^[12–14]^ Intraoperative ultrasound is helpful in locating the FB and determining the surgical approach, and intraoperative ultrasound or radiography can estimate whether the FB has been removed completely.

In addition, once needle injuries have occurred, tetanus should always be considered, especially in children with no, incomplete, or unknown history of immunization and unclean or contaminated wounds.^[17–19]^ Notably, *Stone RK* et al reported a pediatric case with a needle in the liver due to suspected abuse,^[5]^ and *Saitua F* et al reported a case with a needle in the liver that was suspected to be caused by the caregiver’s negligence.^[13]^ Therefore, to prevent the occurrence of intrahepatic needles, the most important measures may be to take full care of children and provide effective education for caregivers and children, in which primary health care institutions or communities may play an important role because of their advantages of coverage and quantity.

## 5. Conclusion

In summary, pediatric cases involving needles in the liver are rare. A history of swallowing and trauma cannot be ignored, and diagnosis and localization depend on imaging, especially CT. In most cases, surgical removal is the first choice of treatment. Moreover, intraoperative localization and confirmation of whether the FB has been removed completely are essential, and whether tetanus treatment should be administered should also be considered. It should not be ignored that caregivers’ care for children is of great value for prevention and timely medical treatment.

### Acknowledgment

We acknowledge the parents of the patients for allowing us to learn and share their stories.

### Author contributions

Conceptualization: Hui-wu Xing.

Data curation: Hui-wu Xing.

Formal analysis: Hui-wu Xing.

Investigation: Chen-yu Yang.

Methodology: Hui-wu Xing.

Project administration: Bing-qian Tan.

Resources: Chen-yu Yang.

Software: Hui-wu Xing.

Supervision: Ming-man Zhang.

Validation: Chen-yu Yang.

Visualization: Hui-wu Xing.

Writing– original draft: Hui-wu Xing.

Writing– review & editing: Ming-man Zhang.
